# Monocyte human leukocyte antigen – Antigen D related, neutrophil oxidative burst and cytokine analysis in patients of decompensated cirrhosis with and without acute-on chronic liver failure

**DOI:** 10.1371/journal.pone.0200644

**Published:** 2018-07-18

**Authors:** Sandeep Satsangi, Ajay Duseja, Meenakshi Sachdeva, Shallu Tomer, Sunil K. Arora, Sunil Taneja, Radha K. Dhiman, Yogesh K. Chawla

**Affiliations:** 1 Department of Hepatology, Postgraduate Institute of Medical Education and Research, Chandigarh, India; 2 Department of Immunopathology, Postgraduate Institute of Medical Education and Research, Chandigarh, India; Hospital Universitari Bellvitge, SPAIN

## Abstract

**Background and aim:**

Due to a dysregulated immune response, patients with acute-on-chronic liver failure (ACLF) have increased risk of infection and multi organ failure in comparison to compensated cirrhosis. The comparative data on the presence of ‘immune paresis’ in patients with ACLF and decompensated cirrhosis without ACLF is not available. Aim of the present study was to compare the immunological parameters in patients with decompensated cirrhosis with and without ACLF.

**Methodology:**

In a prospective study, 76 patients with decompensated cirrhosis with (n = 38) and without (n = 38) ACLF and 10 healthy controls (HC) were evaluated for monocytic human leukocyte antigen–antigen D Related (HLA-DR) expression, mean density of HLA-DR expressed on the surface of these cells, neutrophil oxidative burst (NOB) capacity and serum levels of cytokines (IL-1, IL-6, IL-8, IL10, IL-12, and TNF-α).

**Results:**

Patients of decompensated cirrhosis with and without ACLF demonstrated significantly lower mean percentage of monocytes expressing HLA-DR and quantitative increase in the NOB after stimulation with PMA when compared to HC. However there was no difference in mean percentage of monocytes with HLA-DR expression (43.61±26.56% vs. 43.10±20.98%) (p = 0.91), mean density of HLA-DR expression on the surface (30.34±29.32 vs. 41.71±52.13) (p = 0.42) and quantitative increase in NOB after stimulation with PMA (16.55±11.91 vs. 17.24±16.18) (p = 0.47) amongst patients with decompensated cirrhosis with and without ACLF. Patients with ACLF had significantly higher pro-inflammatory and anti-inflammatory cytokines in comparison to patients with decompensated cirrhosis without ACLF.

**Conclusion:**

Patients with decompensated cirrhosis demonstrate a component of immune-paresis, however there is similar impairment in HLA–DR expression and NOB capacity in patients with and without ACLF. Both inflammatory and anti-inflammatory cytokines are increased in patients with ACLF in comparison to patients with decompensated cirrhosis without ACLF.

## Introduction

Acute-on-chronic liver failure (ACLF) is an acute deterioration of known or unknown chronic liver disease (CLD) and has been defined by the Asia-Pacific Association for the Study of the Liver (APASL) as “acute hepatic insult manifesting as jaundice (defined as serum bilirubin level ≥5 mg/dL) and coagulopathy (define as international normalized ratio ≥1.5), complicated within 4 weeks by ascites and/or encephalopathy in a patient with previously diagnosed or undiagnosed CLD.[1] However this definition does not consider non-hepatic organ failures unlike the American Association for the Study of Liver Diseases (AASLD) and the European Association for the Study of the Liver (EASL) definition.[2]

Even though ACLF is a rapid decompensation of cirrhosis, it differs from patients with decompensated cirrhosis without ACLF [also called chronic liver failure (CLF)] in many aspects. Firstly, the development of hepatic failure and end-organ dysfunction in patients with ACLF is much faster than in patients with decompensated cirrhosis(CLF). Secondly, in ACLF, there exists a potential scope of recovery of liver function (i.e. there is a component of reversibility in ACLF). Thirdly, a well defined precipitating event usually precedes the liver failure in ACLF.[3] Fourthly, unlike patients with CLF, in ACLF there is a component of multi-organ failure and increasing numbers of organ failures (i.e. renal, cerebral, circulatory, and pulmonary) portend progressively worse outcomes. And lastly, patients with ACLF have significantly higher short-term (3-month) mortality than expected with CLF.[4–6]

The immunopathology of ACLF involves alteration in both innate and adaptive immune responses. The innate immune system involving monocytes, dendritic cells (DCs), neutrophils and macrophages are markedly dysfunctional in patients of ACLF. Monocytic human leukocyte antigen–antigen D Related (HLA-DR) expression is known to play a central role in mounting specific immune responses, as they are required for antigen presentation and activation of helper T lymphocytes. A down regulation of the expression of HLA-DR leads to decreased ex vivo production of cytokines following stimulation by lipopolysaccharide (LPS).[7] There are two studies that could demonstrate a reduced ex vivo tumour necrosis factor (TNF)-α secretion and HLA-DR expression on monocytes in patients with ACLF compared to patients with stable liver cirrhosis documenting a sepsis like immune paralysis in patients with ACLF.[7,8]

Neutrophils are known to play a pivotal role in non-specific immune responses and reveal many features which are crucial for organism immunity. Studies conducted in patients of decompensated cirrhosis of liver have shown that NOB is markedly lower in patients of decompensated cirrhosis when compared to healthy controls (HC)[9].

The role of cytokines in ACLF remains a key point in pathogenesis and the cytokine storm involved is closely correlated to disease severity, hepatocyte apoptosis, and mortality.[10]

To the best of our knowledge, no study has compared the immunological parameters of HLA-DR expression, NOB and serum cytokines in decompensated cirrhosis with and without ACLF. The present study was planned to assess the immunological parameters of patients with ACLF and compare them with patients of CLF to ascertain the differences in immunobiology between the two groups.

## Patients and methods

The study was a prospective observational cohort study. Consecutive patients with decompensated cirrhosis of liver admitted in the department of Hepatology, Postgraduate Institute of Medical Education and Research (PGIMER), Chandigarh, India over 14 months were screened. All patients gave a written informed consent and the study was reviewed and had the approval of the institute’s ethics committee, PGIMER, Chandigarh.

### Patients

#### Group 1—Acute-on-chronic liver failure (ACLF)

**Inclusion criteria.** ACLF was defined as per the APASL definition.[1] Variceal bleed was considered as an acute precipitant of ACLF only if it resulted in jaundice and coagulopathy defining ACLF. The diagnosis of underlying CLD was based on previous liver biopsy if available or on clinical, imaging (heterogenous echotexture of liver with irregular outline, altered liver size, or portosystemic collaterals), laboratory (low serum albumin, aspartate aminotransferase/alanine aminotransferase ratio > 1), and endoscopic findings (≥ grade II oesophageal varices).

**Exclusion criteria**. a) Evidence for hepatocellular carcinoma (HCC) or metastatic liver tumor, which could have affected liver function.

b) Patients with evidence of infection at the time of presentation or within 48 hours of admission/presentation were excluded as infection can alter the level of cytokines and affect HLA-DR expression and NOB potential.

Sepsis was defined as the presence of systemic inflammatory response syndrome (SIRS) with definite evidence of bacterial or fungal infection.[11] SIRS was defined by the presence of two or more of the following criteria:

1. Body temperature >38 or <36°C
2. Heart rate >90 per minute
3. Breathing frequency >20 per minute or PaCO2 >4.3 kPa
4. White blood count >12,000 per μl or <4000 per μl or more than 10% immature cells

Patients of ACLF were graded as per Chronic Liver Failure Acute-on-Chronic Liver Failure in Cirrhosis (CANONIC) study criteria to look for number of organ failures as follows.

- No-ACLF included patients with no organ failure or patients with a single non-kidney/non-cerebral organ failure with serum creatinine < 1.5 mg/dL and no hepatic encephalopathy (HE) or patients with single cerebral failure with serum creatinine < 1.5 mg/dL.
- Grade 1 ACLF included patients with single kidney failure or patients with single non-kidney/ non-cerebral organ failure with serum creatinine from 1.5 to 1.9 mg/dL and/or mild to moderate hepatic encephalopathy, or patients with single cerebral failure with serum creatinine from1.5 and 1.9 mg/dL.
- Grades 2 and 3 ACLF included patients with 2 and ≥ 3 organ failures respectively.

**ACLF in patients with unknown CLD**. Some patients presented for the first time as ACLF and were not aware of their underlying chronic liver disease (CLD). In these patients, underlying CLD was diagnosed based on clinical examination, blood investigations, imaging and gastroscopy.

**ACLF in patients with known CLD.** Some patients were known cirrhotics and were on follow up, presented now with ACLF due to a superadded acute insult.

**Definitions of other variables**. Early HE was defined as HE grade I and II whereas advanced HE was defined as HE grade III and IV as per the West Haven criteria. The organ failures (OFs) were defined as per the Chronic Liver Failure Consortium-Organ Failure (CLIF-C OFs) score. Child-Turcotte-Pugh (CTP),[12] Model for End-Stage Liver Disease (MELD),[13] Sequential Organ Failure Assessment Score (SOFA),[14] Acute Physiologic Assessment and Chronic Health Evaluation (APACHE-II),[15] and Chronic Liver Failure Consortium-Acute-on-chronic liver failure (CLIF-C ACLFs),[16] Chronic Liver Failure Consortium-Acute Decompensation (CLIF-C AD)[17] scores were assessed at baseline.

#### Group 2—Chronic liver failure (CLF)

**Inclusion criteria**. Patients were categorized as having CLF if they met the following criteria:

- The diagnosis of cirrhosis was based on previous liver biopsy if available or on clinical examination, imaging (heterogenous echotexture of liver with irregular outline, altered liver size, or portosystemic collaterals), laboratory (low serum albumin, aspartate aminotransferase/alanine aminotransferase ratio > 1), and endoscopic findings (≥ grade II oesophageal varices)
- Decompensation of liver was defined with the presence of ascites, jaundice, hepatic encephalopathy and upper gastrointestinal bleed but not amounting to ACLF as per the APASL criteria or CANONIC criteria.

**Exclusion criteria.** a) Patients with HCC or metastatic liver tumor.

b) Patients with evidence of infection at the time of presentation or within 48 hours of admission/presentation

c) Patients with MELD score ≥25 were excluded to maintain homogeneity of the group.

#### Group 3- Healthy controls (HC)

Ten age matched healthy subjects without a family history of liver disease with normal abdominal ultrasound, normal AST and ALT levels were included as HC. The healthy subjects were selected according to Indian Council of Medical Research (ICMR) guidelines.[18]

### Immunological studies

Three millilitre of blood was drawn from all the patients and HC after an overnight fast. Serum was separated from 2 ml of clotted blood and was stored in -80° C for carrying out cytokine analysis. Mean percentage of monocytes expressing HLA-DR, mean fluorescent intensity (MFI) of HLA-DR expression and quantitative increase in the NOB capacity after stimulation with Phorbol 12-myristate 13-acetate (PMA) was carried out on remaining EDTA-whole blood on the same day.

#### HLA-DR expression on monocytes

Monocyte HLA-DR expression plays a vital role in eliciting specific immune responses and are required for antigen presentation and activation of helper T lymphocytes. 100 μl of whole blood was taken and 2μl of Phycoerythrin (PE) labelled HLA-DR and Fluorescein isothiocyanate (FITC) labelled CD14 antibody was added and incubated in dark at room temperature for 10 min. 2μl of RBC lysis buffer was added, centrifuged for 30sec and incubated in the dark for 10 min. Cells were subsequently centrifuged at 1500rpm for 5 min and washed with 1ml of staining buffer at 1500rpm for 5 min. Cells were resuspended in 300μl of staining buffer, acquired and analysed on flow cytometer (BD FACS Caliber). The quantification of the monocytic HLA-DR was performed using the Cell Quest Pro Software (BD Biosciences, USA). Results were expressed as mean percentage of monocytes expressing HLA-DR and mean fluorescent intensity (MFI) of HLA-DR expression.

#### Neutrophil oxidative burst capacity

Efficient phagocytosis and the subsequent production of reactive oxygen intermediates (ROIs) play an important role in the intracellular killing of microorganisms. The metabolic activity of oxidative burst in vitro and the production of active oxygen compounds can be determined by stimulating the neutrophils with PMA, a protein kinase C activator. 100 μl of blood sample was added in three tubes labelled as stimulated, unstimulated and blood alone. 0.75 μl of Dihydrorhodamine (DHR) 123 was added to all the tubes and incubated for 5min at 37°C. 2 μl of PMA was added only in stimulated tube and incubated for 15min at 37°C. 1ml of FACS lysing solution (BD,USA) was added, incubated for 10 min at 37°C and centrifuged at 1500rpm for 5min. The cells were washed with phosphate buffered solution (PBS) and centrifuged at 1500rpm for 5min. The steps were repeated and cells acquired on flow cytometer (BD Calibur). The formation of reactive oxidants was monitored by the oxidation of dihydrorhodamine 123 to rhodamine. To identify neutrophils, cells were stained with anti–CD16-PE antibody and gated cells were analyzed. Neutrophils were gated using forward and side scatter and the percentage of CD16-positive cells producing reactive oxygen metabolites calculated. Results were expressed as quantitative increase in the NOB after stimulation with PMA (fold change of mean fluorescent intensity).

#### Cytokine bead assay

The quantification of interleukin (IL)-1, IL-6, IL-10, IL-8, IL-12 and tumour necrosis factor (TNF)-α in the whole blood was determined by the human cytometric bead array kit (CBA; BD Bioscience, San Jose, CA, USA) according to the manufacturer’s instructions. Briefly, a vial of lyophilized standard was reconstituted using 0.2ml of assay diluents and allowed to equilibrate for 15 minutes. The beads were Vortexed and 50μl of beads were added to all assay tubes followed by the addition of Human inflammatory cytokine standard dilutions (50μl) and unknown samples (50μl), incubated for 90 min at room temperature in the dark. 1ml of wash buffer was added to each assay tube and centrifuged at 200g for 5 min. The supernatent was discarded leaving 100μl of liquid in each assay tube. 50μl of PE detection reagent was added to all assay tubes and incubated for 90 min in the dark at room temperature. Beads were washed with 1ml. wash buffer at 200g for 5min. The pellet was resuspended in 300μl of wash buffer and acquired on a flow cytometer (BD FACS Aria, BD biosciences U.S.A), and analysed using FCAP software. The results of cytokines are expressed in picograms/millilitre (pg/ml).

Mean percentage of monocytes expressing HLA-DR, mean fluorescent intensity (MFI) of HLA-DR expression and quantitative increase in the NOB after stimulation with PMA (fold change of MFI) was assessed and compared in patients of decompensated cirrhosis with and without ACLF, amongst survivors and non-survivors with ACLF and HC. Serum levels of cytokines were assessed only in patients of decompensated cirrhosis with and without ACLF and not in the healthy controls.

### Statistical analysis

All statistical analysis was performed using SPSS software (version 22, SPSS Inc., Chicago, IL, USA) Mean and standard deviation was used for continuous variables whereas frequency and percentages was used for discrete data. For comparison of frequencies, Chi Square Test was used. For comparison of continuous variables, non parametric (Mann-Whitney U test) unpaired t test was used. Pearson's coefficient was used to test correlations between immunologic parameters and various baseline characteristics. For graphical representation of comparison between two groups GraphPad Prism 6.0 (GraphPad Software, Inc., San Diego, CA) was used. p value was set at a significance of 0.05.

## Results

**Fig 1:**
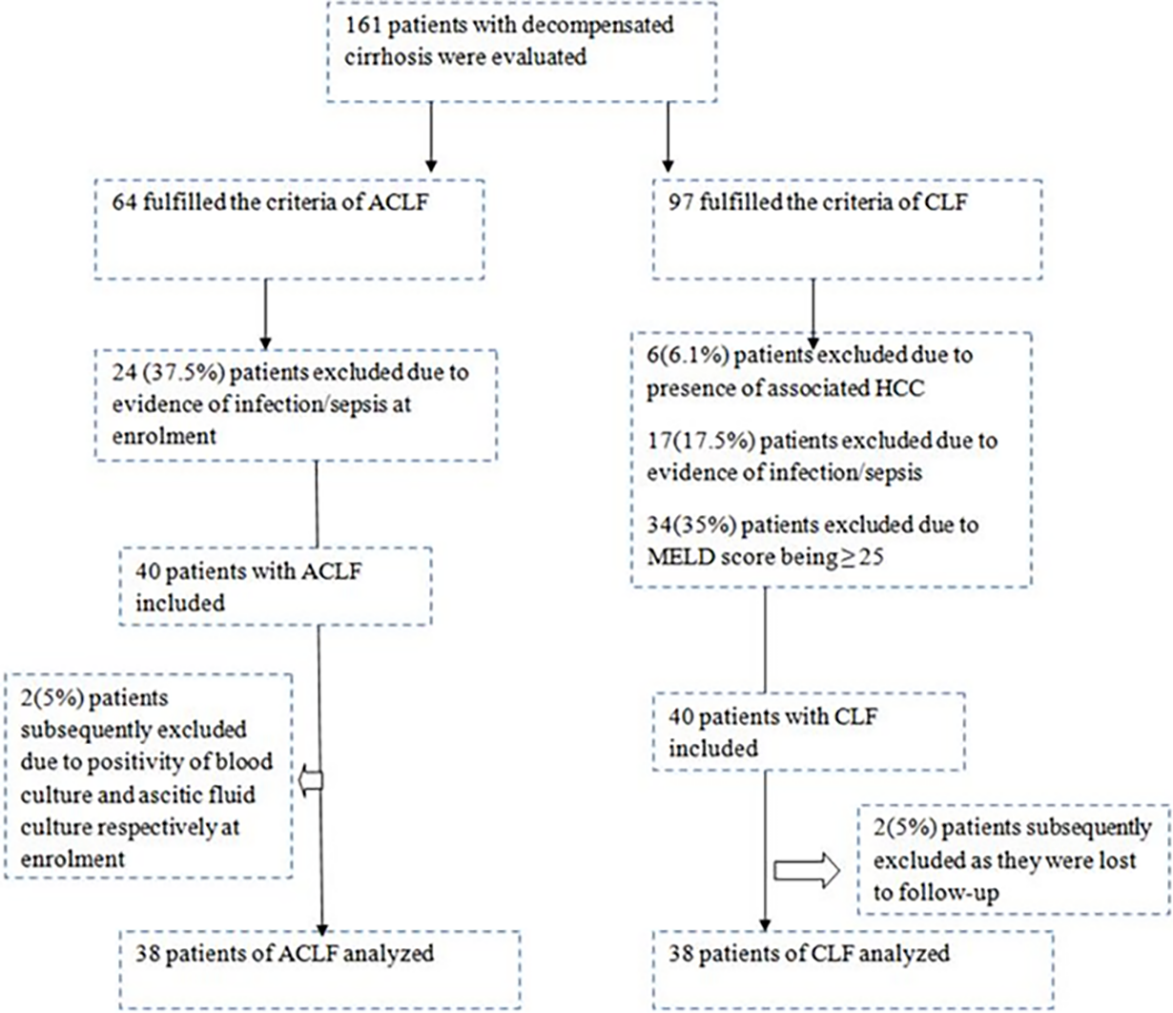
Flow diagram showing the inclusion and exclusion of patients in both groups.

One hundred and sixty one patients with decompensated cirrhosis were evaluated in the study period of which 64 fulfilled the criteria for ACLF and 97 fulfilled the criteria of CLF. Twenty six patients were excluded from the ACLF group and 59 patients were excluded from the CLF group and 76 patients (38 in each group) satisfying inclusion and exclusion criteria were finally included. (Fig 1)

### ACLF group

Thirty five (92.1%) of 38 patients with ACLF were male with a mean age of 45.47±12.95 years. All patients with ACLF had cirrhosis of liver as underlying CLD. The commonest etiology of the acute precipitating insult and underlying CLD was alcohol in 27(71.05%) and 28 (73.68%) patients respectively. As per the CANONIC grading system, 4(10.52%) patients had no-ACLF, whereas ACLF grade 1, 2 and 3 was present in 8(21.05%), 14(36.84%) and 12(31.57%) patients respectively. Among 38 patients with ACLF, 13(34.21%) expired by 28 and 17 (44.73%) by 90 days. Clinical profile, hematological, and other laboratory parameters of 38 patients with ACLF are shown in Table 1.

**Table 1:** Showing the baseline characteristics and comparison between the patients with known and unknown CLD in ACLF.

**Parameters**	**Underlying CLD**			**p -value**
	Total(38)	Unknown(26)(68.42%)	Known(12)(31.57%)	
**Age,years ± SD, (y)**	45.47±12.95	46.88±13.72	42.42±11.00	0.330
**Males (%)**	35 (92.10%)	24(92.30%)	11(91.66%)	0.946
**Early HE**	18(47.36%)	13(50%)	5(41.66%)	0.632
**Advanced HE**	9(23.68%)	6(23.07%)	3(25%)	0.897
**Mean duration of illness at presentation(Days)**	28.97±15.68	23.31±10.59	41.25±18.23	**<0.0001**
**Past history of Jaundice**	10(26.31%)	0	10(83.33%)	**<0.0001**
**Hb,g/dl**	9.37±2.09	9.49±2.11	9.18±2.11	0.606
**Total leukocyte count, per mm^3^**	13,537±8002	13,297±7480	14058±9370	0.789
**Platelets (× 10^3^/mm^3^)**	118.26±72.93	122.65±78.15	108.75±62.18	0.592
**International normalized ratio**	2.29±0.75	2.17±0.71	2.54±0.79	0.157
**Urea, mg/dl**	71.76±55.06	74.73±60.34	65.33±43.07	0.631
**Creatinine, mg/dl**	2.23±1.27	2.21±1.23	2.25±1.40	0.930
**Na,mmol/L**	133.45±7.50	134.62±7.66	130.92±6.74	0.161
**Bilirubin,mg/dl**	23.08±8.72	21.83±8.51	25.77±8.95	0.200
**AST,U/L**	162.63±95.91	172.69±105.73	140.83±69.10	0.348
**ALT,U/L**	113.82±158.71	135.15±188.30	67.58±27.14	0.227
**Albumin,g/dl**	2.65±0.57	2.73±0.56	2.50±0.59	0.260
**Etiology (acute hepatic insult)**				0.449
Hepatitis E virus	3(7.89%)	2(7.69%)	1(8.33%)	
Hepatitis A virus	1(2.63%)	1(3.84%)	0	
HBV reactivation	0	0	0	
Alcohol	27(71.05%)	19(73.07%)	8(66.66%)	
Drugs (antitubercular)	2(5.26%)	2(7.69%)	0	
Autoimmune hepatitis flare	1(2.63%)	0	1(8.33%)	
Variceal bleed	1(2.63%)	0	1(8.33%)	
Unknown	3(7.89%)	2(7.69%)	1(8.33%)	
**Etiology (chronic liver disease)**				0.523
Hepatitis B virus	1(2.63%)	1(3.84%)	0	
Hepatitis C virus	1(2.63%)	1(3.84%)	0	
Alcohol	28(73.68%)	18(69.23%)	10(83.33%)	
NASH	2(5.26%)	2(7.69%)	0	
Autoimmune hepatitis	3(7.89%)	1(3.84%)	2(16.66%)	
Wilsons	0	0	0	
HVOTO	0	0	0	
Hepatitis C + Alcohol	1(2.63%)	1(3.84%)	0	
Cryptogenic	2(5.26%)	2(7.69%)	0	
**CTP**	11.76±1.38	11.58±1.39	12.17±1.33	0.227
**MELD**	32.53±6.76	32.00±7.07	33.67±6.19	0.488
**CLIF-C-OFs**	11.47±2.43	11.04±2.14	12.42±2.84	0.106
**CLIF-C-ACLF**	53.44±9.44	52.91±8.44	54.55±11.65	0.645
**SOFA**	8.97±3.08	8.62±2.83	9.75±3.57	0.297
**APACHE-II**	21.42±6.40	20.85±6.11	22.67±7.11	0.423
**28-day mortality**	13(34.21%)	7(26.92%)	6(50%)	0.163
**90 -day mortality**	17(44.73%)	9(34.61%)	8(66.66%)	0.65

ALT: Alanine aminotransferase; AST: Aspartate aminotransferase

### CLF group

Twenty nine (76.31%) of 38 patients with CLF were male with a mean age of 48.24 ± 13.23 years. The etiology of cirrhosis was alcohol, NASH, and autoimmune hepatitis in 15(39.47%), 8(21.05%), and 4(10.52%) patients, respectively. Twenty two (57.9%) patients had CTP class B cirrhosis whereas 16 (42.1%) patients had class C cirrhosis. Clinical profile, hematological, and other laboratory parameters of 38 patients with CLF are shown in Table 2.

**Table 2:** Baseline characteristics of patients with Chronic liver failure (CLF).

**Parameters**	**Total—38**
**Age,years ± SD, (y)**	48.24±13.23
**Males (%)**	29 (76.31%)
**Hepatic encephalopathy(%)**	2(5.26%)
Early (%)	1(50%)
Advanced (%)	1(50%)
**History of UGI bleed (%)**	6(15.78%)
**History of Jaundice (%)**	17(44.7%)
**Ascites (%)**	33(86.84%)
Grade 1 (%)	7(21.21%)
Grade 2 (%)	15(45.45%)
Grade 3 (%)	11(33.33%)
**Hb, g/dl**	9.26±2.49
**Total leukocyte count, per mm3**	6697.37±3108.87
**Platelets (× 10^3^/mm3)**	91.26±62.84
**International normalized ratio**	1.48±0.32
**Urea, mg/dl**	49.42±42.28
**Creatinine, mg/dl**	1.06±0.47
**Na, mmol/L**	133.47±7.20
**Bilirubin,mg/dl**	3.45±3.25
**AST,U/L**	71.13±41.38
**ALT,U/L**	36.13±29.00
**Albumin,g/dl**	2.59±0.68
**Etiology of cirrhosis**	
Hepatitis B virus	1(2.63%)
Hepatitis C virus	4(10.52%)
Alcohol	15 (39.47%)
Alcohol + Hepatitis C virus	1(2.63%)
NASH	8(21.05%)
Autoimmune hepatitis	4(10.52%)
Wilson’s	1(2.63%)
Budd Chiari Syndrome	1(2.63%)
Cryptogenic	3(7.89%)
**CTP**	8.95±1.73
Class B	22 (57.9%)
Class C	16 (42.1%)
**MELD**	15.7±5.05
**3 month mortality**	2(5.3%)

ALT: Alanine aminotransferase; AST: Aspartate aminotransferase; BCS: Budd Chiari syndome; NASH: Non-alcoholic steatohepatitis

### Immunologic parameters

#### I] Immunologic parameters in patients with ACLF, CLF and HC

**Fig 2:**
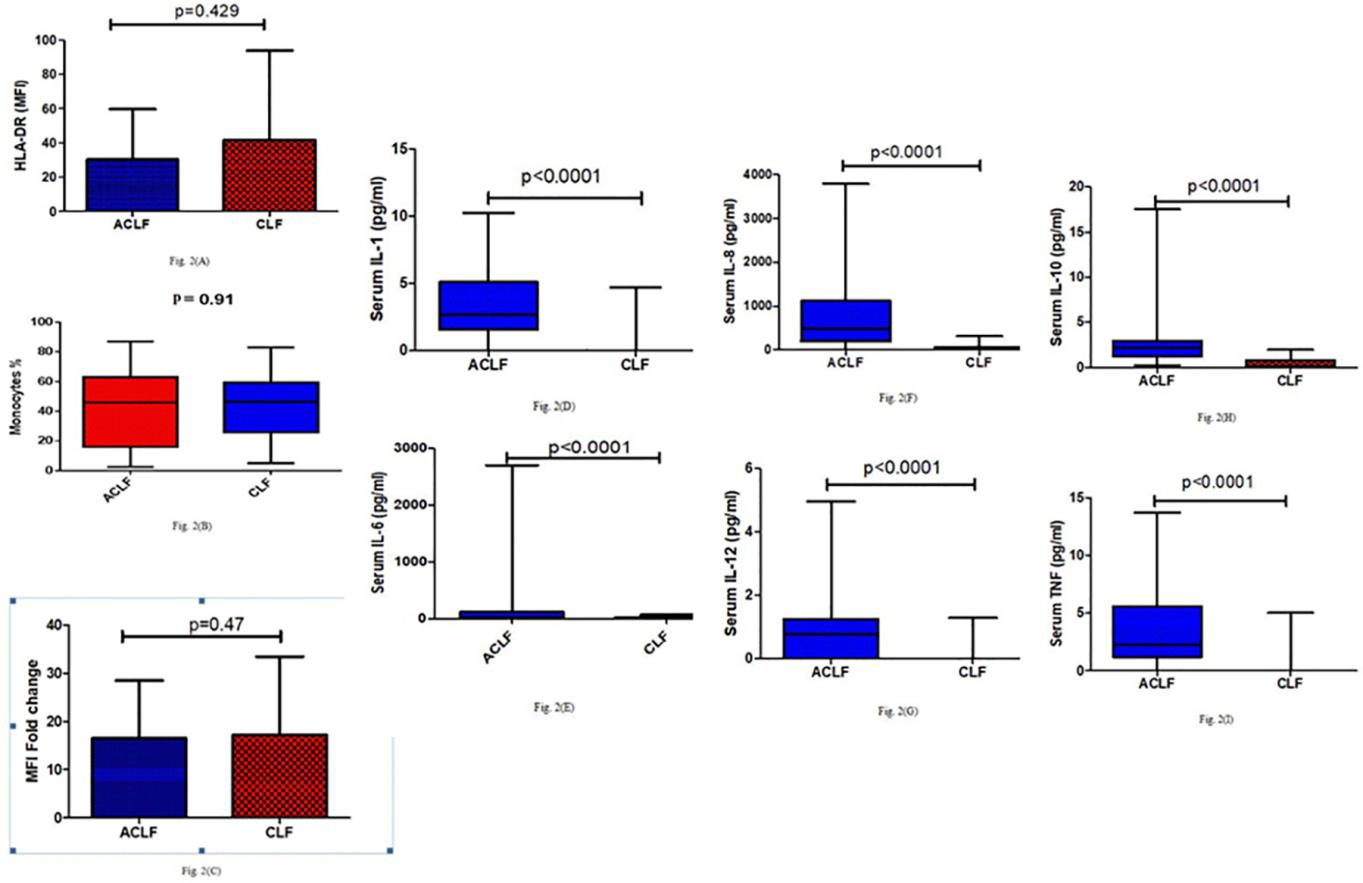
Immunologic parameters in patients with ACLF and CLF. **(A):** Mean fluorescent intensity (MFI) of HLA-DR expression in patients with ACLF and CLF. **(B):** Mean percentage of monocytes expressing HLA-DR in patients with ACLF and CLF. **(C):** Quantitative increase in the neutrophilic oxidative burst (MFI fold change) after stimulation with PMA between ACLF and CLF. **(D):** Serum IL-1 levels in patients with ACLF and CLF. **(E):** Serum IL-6 levels in patients with ACLF and CLF. **(F):** Serum IL-8 levels in patients with ACLF and CLF. **(G):** Serum IL-12 levels in patients with ACLF and CL**F. (H):** Serum IL-10 levels in patients with ACLF and CLF. **(I):** Serum TNF-α levels in patients with ACLF and CLF.

Patients of both ACLF and CLF demonstrated lower mean percentage of monocytes expressing HLA-DR, mean density of HLA-DR expression on the surface of these cells and quantitative increase in the NOB after stimulation with PMA when compared to HC.(Table 3, S1–S4 Tables). However, there was no significant difference in mean percentage of monocytes expressing HLA-DR (43.61±26.56% vs. 43.10±20.98%, p = 0.91), mean density of HLA-DR expression on the surface of these cells (30.34±29.32 vs. 41.71±52.13, p = 0.42) and quantitative increase in the NOB after stimulation with PMA (16.55±11.91 vs. 17.24±16.18, p = 0.47) in patients of ACLF and CLF. (Fig 2A–2I)

**Table 3:** Immunological parameters in patients of ACLF, CLF and HC.

**Parameter**	**ACLF(n = 38)**	**CLF(n = 38)**	**HC (n = 10)**	**ACLF vs. CLF**	**ACLF vs. HC**	**CLF vs. HC**
				p value		
**HLA-DR expression (MFI)**	30.34±29.32	41.71±52.13	50.60±13.96	0.42	**0.04**	0.59
**Mean percentage of monocytes expressing HLA-DR**	43.61±26.56%	43.10±20.98%	77.80±3.55%	0.91	**<0.0001**	**<0.0001**
**Quantitative increase in the NOB**	16.55±11.91	17.24±16.18	50.39±45.98	0.47	**<0.0001**	**0.001**
**IL-1 (pg/ml) ±SD**	3.50±2.67	0.35±1.12	**-**	**<0.0001**	**-**	**-**
**IL-10 (pg/ml) ±SD**	3.28±3.68	0.45±0.62	**-**	**<0.0001**	**-**	**-**
**TNF-α (pg/ml) ±SD**	3.27±2.94	0.35±1.12	**-**	**<0.0001**	**-**	**-**
**IL-6 (pg/ml) ±SD**	220.9±611.2	16.51±16.52	**-**	**<0.0001**	**-**	**-**
**IL-8 (pg/ml) ±SD**	781.7±865.1	48.85±62.06	**-**	**<0.0001**	**-**	**-**
**IL-12 (pg/ml) ±SD**	0.98±1.14	0.10±0.27	**-**	**<0.0001**	**-**	**-**

HLA-DR: Human Leukocyte Antigen—antigen D Related, MFI: Mean fluorescent intensity, NOB: Neutrophilic oxidative burst, IL: Interleukin

Patients with ACLF had significantly higher levels of pro-inflammatory (pg/ml) [IL1β (3.5±2.6 vs. 0.35±1.12, p = <0.0001), IL-6(220.9±611.2 vs. 16.51±16.52, p<0.0001), IL-8 (781.7±865.1 vs. 48.85±62.06, p<0.0001), IL-12 (0.9±1.1 vs. 0.10±0.27, p<0.0001), TNFα (3.2±2.9 vs. 0.35±1.12, p <0.0001) and anti-inflammatory (pg/ml) [IL-10(3.2±3.6 vs.0.4±0.6, p = <0.0001] cytokines in comparison to patients with CLF. (Table 3)

#### II] Immunologic parameters in survivor and non-survivor patients amongst ACLF

The mean percentage of monocytes expressing HLA-DR was significantly lower in the non-survivors (21.32 ±16.77%) when compared to survivor (61.67± 16.07%) group with ACLF (P<0.0001) however the mean density of HLA-DR expression and the quantitative increase in the neutrophilic oxidative burst after stimulation with PMA was insignificantly different between the two groups. Patients with ACLF in the non-survivor group had significantly higher pro-inflammatory (pg/ml) [IL1β (5.2 ±2.6 vs. 2.1±1.7, p = <0.0001), IL-6 (479.78± 877.5 vs. 20.09±15.74, p = <0.0001), IL-8 (1116.0±946.8 vs. 410.3 ±494.0, p = 0.0006), IL-12 (1.61±1.37 vs. 0.48±0.55, p = 0.002), TNF- α (5.29±3.07 vs. 1.64±1.48, p <0.0001) and anti-inflammatory (pg/ml) [IL-10 (5.02±4.91 vs. 1.70±0.88 p = 0.002) cytokines in comparison to patients in the survivor group. (Table 4)

**Table 4:** Immunological parameters in patients in the survivor and non-survivor group with ACLF.

Parameter	ACLF survivors (n = 21)	ACLF non-survivors (n = 17)	p value
**HLA-DR expression (MFI)**	36.59±32.30	22.61±23.87	0.14
**Mean percentage of monocytes expressing HLA-DR(%)**	61.67± 16.07	21.32 ±16.77	**<0.0001**
**Quantitative increase in the NOB**	17.29±13.29	15.63±10.28	0.67
**IL-1 (pg/ml) ±SD**	2.1±1.76	5.2±2.6	**<0.0001**
**IL-10 (pg/ml) ±SD**	1.70±0.88	5.02±4.91	**0.002**
**TNF-α (pg/ml) ±SD**	1.64±1.48	5.29±3.07	**<0.0001**
**IL-6 (pg/ml) ±SD**	20.09±15.74	479.78 ± 877.5	**<0.0001**
**IL-8 (pg/ml) ±SD**	410.3±494.0	1116.0±946.8	**0.0006**
**IL-12 (pg/ml) ±SD**	0.48±0.55	1.61±1.37	**0.002**

## Discussion

ACLF is a distinct clinical entity which is different from patients with decompensated cirrhosis without ACLF in view of the rapid downhill course occurring in a patient with known or unknown CLD and because of potential reversibility of the acute event.[19] The evidence pointing to the concept of 'immune paralysis' in patients with ACLF has gained a lot of interest recently, however a clear distinction in the immunologic profile in patients of decompensated cirrhosis with and without ACLF is lacking. Ours is probably the first study, comparing the immunological parameters in patients of decompensated cirrhosis with and without ACLF and demonstrating an equivalent innate immunologic dysfunction in both the groups of patients, however ACLF was associated with a cytokine storm involving both pro- and anti-inflammatory cytokines.

The expression of major histocompatibility complex (MHC) class II on monocytes is a vital pre requisite for adequate immune function.[20] Several initial studies have shown that HLA-DR expression on monocytes decreases after surgery, major trauma and organ transplantation.[21] Wasmuth et al.[7] in their study assessed the immunological parameters in patients with ACLF, severe sepsis and compensated cirrhosis. The authors demonstrated a significant decrease of HLA-DR expression in patients with ACLF and severe sepsis when compared to patients with compensated cirrhosis. Patients with ACLF demonstrated a sepsis like immune paralysis as ex vivo secretion of TNF-α after stimulation with LPS was reduced in patients with ACLF and severe sepsis when compared to patients with compensated cirrhosis. This study demonstrated that the magnitude of cellular immune depression is similar in patients with ACLF and severe sepsis. In our study, we have demonstrated significantly lower mean percentage of monocytes expressing HLA-DR in patients with both ACLF and CLF when compared to HC and noted similar immune dysfunction in patients of decompensated cirrhosis with or without ACLF as there was no significant difference demonstrated in either the mean percentage of monocytes expressing HLA-DR or quantitative expression of HLA-DR on the surface of these cells between both the groups of patients (p>0.05).

In an elegant study done by Xing et al.[8] gradually falling percentage of HLA-DR expression was demonstrated in patients with chronic hepatitis B, liver cirrhosis and different stages of ACLF compared with healthy controls (84·1 ± 6·5%, 65·7 ± 11·8%, 53·2 ± 8·7% and 38·9 ± 9·3% *vs*. 93·5 ± 2·4%; *p*< 0·001). The authors also demonstrated a lower monocyte HLA-DR expression in patients who did not survive when compared to those who survived with ACLF (P<0.01). In our study we have demonstrated a significant reduction in the mean percentage of monocytes expressing HLA-DR in non-survivor patients of ACLF when compared to those who survived, indicating a possible link of immune paralysis to increased mortality in these patients.

In a study by Panasiuk et al.[9] in patients with decompensated cirrhosis (without ACLF), NOB and neutrophilic phagocytic capacity was assessed. Markedly lower production of ROS was observed in patients with liver cirrhosis when compared to healthy controls after PMA stimulation (MFI: 42.7±14.6 *vs*. 50.2±13.3, *p*<0.01).[9] Patients with CTP class C cirrhosis demonstrated lower MFI of NOB after PMA stimulation when compared to patients with CTP class B cirrhosis (41.1±12.6 vs. 44.3±10.1). The authors demonstrated phagocytizing neutrophils to have markedly lower metabolic potential in patients with liver cirrhosis (LC) when compared to HC. No direct comparison of neutrophil functions in patients of decompensated cirrhosis with and without ACLF exists in literature. In our study, we have assessed the quantitative increase in the NOB activity after stimulation with PMA and have noted significant lower increase in NOB in patients of both ACLF and CLF when compared to HC, however similar impairment of NOB was noted in patients of decompensated cirrhosis with or without ACLF.

In a study by Mao et al.[22] one hundred forty-nine patients with ACLF were enrolled and cytokine levels such as interferon-γ (IFN-γ), IL-10, IL-4, IL-2, and TNF-α were detected on admission and on days 7, 14, 21, and 30 during hospitalization and compared with healthy controls. It was shown that the levels of all the cytokines measured were significantly higher in the ACLF group when compared to healthy controls. The authors also postulated that decreasing the cytokine levels by plasmapheresis (PP) would improve the survival rate in patients with ACLF. After 30 days of treatment with PP, it was demonstrated that the levels of all the cytokines significantly decreased in patients who received PP and the survival rate at day 30 was significantly higher in patients who received PP vs. those who received only standard medical treatment (46.7% vs. 28.7%, p<0.05).

In a recent study done by Claria et al.[23] a systemic inflammation (SI) hypothesis was proposed, suggesting ACLF to be an acute exacerbation of the SI already present in decompensated cirrhosis. The authors demonstrated that patients presenting with ACLF exhibited significantly higher levels of pro and anti-inflammatory cytokines than patients without ACLF. Patients with decompensated cirrhosis without ACLF also demonstrated significantly higher levels of cytokines when compared to HC. The results of our study also demonstrate a significant increase of pro-inflammatory cytokines in patients of ACLF when compared to CLF and also show that patients who did not survive had higher levels of pro-inflammatory cytokines when compared to those who survived with ACLF. This data clearly highlights the fact that patients with ACLF have higher pro-inflammatory cytokines which might cause tissue injury leading to a greater severity of liver disease which in turn results in higher mortality.

The strengths of our study include (i) an exhaustive evaluation of the immunological profile in patients of decompensated cirrhosis with and without ACLF in whom infection has been excluded (ii) a direct comparison of the immunological parameters in patients with ACLF and CLF (iii) demonstrating a component of immune paralysis equivalent to that of ACLF in patients of CLF (iv) demonstrating a higher degree of immune paralysis with lesser percentage of monocytes expressing HLA-DR in patients who did not survive when compared to those who survived with ACLF. The limitations of our study include a small sample size and the lack of serial evaluation of immunologic profile in patients with ACLF and CLF after admission.

In conclusion, our study demonstrates that patients with decompensated cirrhosis have a component of immune-paresis, but the impairment in HLA–DR expression and NOB capacity is similar in patients with and without ACLF. In addition, both pro and anti-inflammatory cytokines are increased in patients with ACLF in comparison to CLF.

## Supporting information

S1 Table ACLF and HC group statistics. (DOCX)

S2 Table Independent Samples t Test comparing the means of ACLF and HC. (DOCX)

S3 Table CLF and HC group statistics. (DOCX)

S4 Table Independent Samples t Test comparing the means of CLF and HC. (DOCX)
