# Supplementary material for: Monocyte human leukocyte antigen – Antigen D related, neutrophil oxidative burst and cytokine analysis in patients of decompensated cirrhosis with and without acute-on chronic liver failure
Source: PLoS One. 2018 Jul 18;13(7):e0200644. doi: 10.1371/journal.pone.0200644 (PMC6051623; doi:10.1371/journal.pone.0200644)
Supplement: S1 Table — (DOCX) [file pone.0200644.s001.docx]

ACLF vs. Healthy Controls

| **Group Statistics** | | | | | |
| --- | --- | --- | --- | --- | --- |
|  | Sample | N | Mean | Std. Deviation | Std. Error Mean |
| HLA DR | Test | 38 | 30.3421 | 29.32947 | 4.75787 |
|  | Control | 10 | 50.6030 | 16.12055 | 5.09776 |
| Monocytes % | Test | 38 | 43.618 | 25.9686 | 4.2127 |
|  | Control | 10 | 77.792 | 3.8414 | 1.2148 |
| DHR (MFI) Fold change | Test | 38 | 16.5539 | 11.91640 | 1.93310 |
|  | Control | 10 | 50.3930 | 45.98207 | 14.54081 |
