# Supplementary material for: Monocyte human leukocyte antigen – Antigen D related, neutrophil oxidative burst and cytokine analysis in patients of decompensated cirrhosis with and without acute-on chronic liver failure
Source: PLoS One. 2018 Jul 18;13(7):e0200644. doi: 10.1371/journal.pone.0200644 (PMC6051623; doi:10.1371/journal.pone.0200644)
Supplement: S2 Table — (DOCX) [file pone.0200644.s002.docx]

ACLF vs. Healthy Controls

| **Independent Samples Test** | | | | |
| --- | --- | --- | --- | --- |
|  | | t-test for Equality of Means | | |
|  |  | t | df | Sig. (2-tailed) |
|  |  |  |  |  |
| HLA DR | Equal variances assumed | -1.563 | 46 | .125 |
|  | Equal variances not assumed | -2.171 | 26.599 | .039 |
| Monocytes % | Equal variances assumed | -4.118 | 46 | .000 |
|  | Equal variances not assumed | -7.795 | 42.209 | .000 |
| DHR (MFI) Fold change | Equal variances assumed | -4.144 | 46 | .000 |
|  | Equal variances not assumed | -2.307 | 9.320 | .046 |
