# Supplementary material for: Monocyte human leukocyte antigen – Antigen D related, neutrophil oxidative burst and cytokine analysis in patients of decompensated cirrhosis with and without acute-on chronic liver failure
Source: PLoS One. 2018 Jul 18;13(7):e0200644. doi: 10.1371/journal.pone.0200644 (PMC6051623; doi:10.1371/journal.pone.0200644)
Supplement: S4 Table — (DOCX) [file pone.0200644.s004.docx]

CLF vs. Healthy Controls

| **Independent Samples Test** | | | | |
| --- | --- | --- | --- | --- |
|  | | t-test for Equality of Means | | |
|  |  | t | df | Sig. (2-tailed) |
|  |  |  |  |  |
| HLA DR | Equal variances assumed | -.224 | 46 | .824 |
|  | Equal variances not assumed | -.382 | 44.579 | .594 |
| Monocytes % | Equal variances assumed | -5.164 | 46 | .000 |
|  | Equal variances not assumed | -9.595 | 44.083 | .000 |
| DHR (MFI) Fold change | Equal variances assumed | -3.733 | 46 | .001 |
|  | Equal variances not assumed | -2.244 | 9.594 | .050 |
